# Herb-Induced Liver Injury by Ayurvedic Medicine With Severe Lactic Acidosis: A Case Report

**DOI:** 10.7759/cureus.34761

**Published:** 2023-02-08

**Authors:** Deepak S Sharma, Ahmed Ahmed, Ali A Razak, Priyanka Sharma

**Affiliations:** 1 Critical Care Medicine, Sandwell and West Birmingham Hospitals NHS Trust, Birmingham, GBR; 2 Anesthesia and Critical Care Medicine, Sandwell and West Birmingham Hospitals NHS Trust, Birmingham, GBR; 3 Research and Development, University of Birmingham, Birmingham, GBR

**Keywords:** critical care and hospital medicine, herbal-induced liver injury (hili), drug-induced liver injury (dili), rucam score, ayurvedic medicine

## Abstract

Lactate is the basic blood parameter in the arsenal of an intensivist when managing a critically ill patient. A 62-year-old male presented with nausea and vomiting. He had been using an Ayurvedic medication, Insulin Management Expert (IME-9), for his type 2 diabetes mellitus and was found to have severe lactic acidosis that was resistant to initial fluid resuscitation and Ayurvedic medicine-induced liver injury. He required admission to critical care for organ support and ultimately recovered. Because current literature on the adverse effects of this Ayurvedic medication, particularly hepatotoxicity, is limited, causality was determined using the adverse drug association tool Roussel Uclaf Causality Assessment Method (RUCAM), which determined this as a probable cause with a strong score of seven. As a result, our case adds a vital gear to the wheel of current research literature.

## Introduction

According to current literature, elevated lactate and poor lactate clearance are associated with poor prognosis in critical care medicine [1]. The conventional differentials for elevated lactate in critically ill patients are sepsis, hypoperfusion, or regional ischemia; however, alternative causes of elevated lactate are frequently overlooked despite being equally important and warranting further investigation. Our case illustrates a very unusual case of lactic acidosis caused by the use of Ayurvedic medication. Careful history-taking and ruling out the differential diagnosis of various other causes of lactic acidosis, as well as an in-depth understanding of lactic acidosis physiology, are essential for treating such patients and avoiding the assumption of labeling them as having an inappropriately poor prognosis.

## Case presentation

A 62-year-old man with a history of type 2 diabetes mellitus was admitted to the intensive care unit after experiencing nausea and vomiting for two days but no trauma, convulsions, or infectious symptoms. He was a non-smoker and drank no alcohol. A thorough medication history revealed that he had not taken his metformin for approximately two weeks and believed that these medications did not work; instead, he had been taking a herbal medication known as Insulin Management Expert (IME-9) for approximately 25 days which he used to take two tablets three times a day, 30 minutes before meals. His clinical exam revealed mild tachycardia (117 beats per minute), normal blood pressure, diffuse abdominal tenderness with no guarding, and no icterus. The rest of his exam was uneventful, with no signs of chronic liver or kidney disease.

Investigations

His bloodwork initially revealed severe metabolic acidosis, with lactate levels that were too high to measure using arterial blood gas (ABG) analysis. Formal blood tests provided to the biochemistry department revealed a lactate level of 25.02 mmol/L as well as abnormally high transaminitis but normal albumin levels and a normal prothrombin time (Table 1). The toxicology screen, which included paracetamol, salicylate, and alcohol levels, was within normal limits. His abdominal imaging revealed no mesenteric ischemia, occlusion, or significant bowel stenosis.

**Table 1:** Trend of blood results and rest of work-up eGFR- estimated glomerular filtration rate; INR- international normalised ratio; PaO2- partial pressure of oxygen; RRT - renal replacement therapy

	Results on admission	Results after 6 hours of fluid resuscitation	Results after 12 hours of RRT	Results on discharge from ICU	Results on discharge from hospital
White blood cell count	16.5 x10^9^/L	18 x10^9^/L	11.7 x10^9^/L	5.4 x10^9^/L	6.8 x10^9^/L
Haemoglobin level	160 g/L	128 g/L	123 g/L	124 g/L	115 g/L
Platelet count	116 x10^9^/L	109 x10^9^/L	155 x10^9^/L	52 x10^9^/L	114 x10^9^/L
Sodium	138 mmol/L	137 mmol/L	138 mmol/L	138 mmol/L	138 mmol/L
Potassium	4.3 mmol/L	5.3 mmol/L	3.4 mmol/L	3.8 mmol/L	3.5 mmol/L
Creatinine	100 micromol/L	115 micromol/L	72 micromol/L	58 micromol/L	55 micromol/L
Urea	7.6 mmol/L	8.6 mmol/L	5.1 mmol/L	2.6 mmol/L	3.5 mmol/L
eGFR	>90 mL/min/1.73m^2^	>90 mL/min/1.73m^2^	>90 mL/min/1.73m^2^	>90 mL/min/1.73m^2^	>90 mL/min/1.73m^2^
Phosphate	-	2.06 mmol/L	0.47 mmol/L	0.59 mmol/L	0.79 mmol/L
Adjusted calcium	-	-	2.33 mmol/L	2.37 mmol/L	2.16 mmol/L
Magnesium	-	0.81 mmol/L	0.71 mmol/L	0.89 mmol/L	0.86 mmol/L
Blood ketones	0.1 mmol/L	0.2 mmol/L	-	0.1 mmol/L	-
Alanine transaminase	1100 U/L	1030 U/L	800 U/L	207 U/L	37 U/L
Aspartate transaminase	800 U/L	601 U/L	635 U/L	159 U/L	-
Alkaline phosphatase	180 U/L	176 U/L	64 U/L	75 U/L	-
Bilirubin	38 micromol/L	32 micromol/L	20 micromol/L	21 micromol/L	15 micromol/L
Albumin level	39 g/L	33 g/L	30 g/L	26 g/L	23 g/L
INR	-	1.52	-	2.18	1.50
Prothrombin time	-	18.1 s	-	26.4 s	18 s
Creatine kinase	317 U/L	-	-	25 U/L	-
pH	7.239	7.238	7.418	7.437	7.195
PaO2	18 kilopascals	11 kilopascals	15 kilopascals	16 kilopascals	12 kilopascals
Base excess	-14.1 mmol/L	-18.5 mmol/L	-0.6 mmol/L	1.9 mmol/L	-10.2 mmol/L
Lactate	Un-recordable	Un-recordable	3.9 mmol/L	3.0 mmol/L	5.2 mmol/L
Glucose	7.1 mmol/L	11.9 mmol/L	11.6 mmol/L	13.0 mmol/L	11.5 mmol/L
Blood lactate (Lab)	-	25.02 mmol/L	-	-	-
C-reactive protein (CRP)	33 mg/L	43 mg/L	52 mg/L	47 mg/L	11 mg/L
Paracetamol level	<10 mg/L				
Salicylate level	127 mg/L	72 mg/L			
Urine toxicology	Negative for cocaine, amphetamine, MDMA, benzodiazepines, cannabis, morphine				
Urine creatinine	3.2 mmol/L				
Urine alcohol level	<100 ml/L				
Electrocardiography	Nil significance except for sinus tachycardia				
Echocardiogram	No abnormalities				
Kidney, liver immunology & virology screen	No significant findings				
Microbiology results	No growth of any organisms from sputum, blood, and urine samples				
CT thorax, abdomen, and pelvis with contrast	No significant abnormality identified to account for the patient's symptoms				

Differential diagnosis

We ruled out common causes of hyperlactatemia, such as sepsis, tissue hypoperfusion, regional ischemia, and cardiogenic shock. Imaging was performed in tandem to rule out obstructive shock, mesenteric ischemia, and any significant cancer. During interrogation, there was no strong indication of seizures, strenuous exertion, or shivering. His ketones were normal. There were no signs of carbon monoxide, cyanide, iron, or any other chemical overdose or poisoning. Other potential causes of liver disease were thoroughly investigated, including various hepatitis viruses, Epstein-Barr virus (EBV), cytomegalovirus (CMV), and varicella-zoster virus (VZV); autoimmune hepatic conditions; and hepatic vascular diseases such as Budd-Chiari syndrome and sinusoidal obstruction. He had no previous hospitalizations in the previous six months.

Treatment

Fluid resuscitation was performed using four liters of intravenous 0.9% saline guided by focused ultrasound and other usual measures for predicting fluid responsiveness; however, a repeat ABG after six hours revealed unrecordable lactate (Table 1). Continuous renal replacement therapy (CRRT) was started due to refractory metabolic acidosis with severe hyperlactatemia and the suspected use of herbal drugs. The toxicology team and regional tertiary liver center were involved in the further management of this suspected unknown intoxication with multi-organ involvement. Vitamin K and phosphate replacements were given to the patient. With CRRT, his lactate levels dropped substantially, dropping to 2.6 in less than 12 hours, and it was later discontinued after 24 hours when his blood results and base deficit improved.

Outcome and follow-up

The patient improved after three days of critical care and was stepped down to the medical gastroenterology ward. His liver function tests (LFTs) improved while he was under their care. Following adequate electrolyte replenishment and blood sugar control, the patient was discharged after a total of 10 days in the hospital with pre-booked follow-up with his general practitioner to monitor his LFTs.

## Discussion

Lactate is a by-product of glycolysis that is produced in the central nervous system, erythrocytes, skin, and skeletal muscles. In the presence of adequate oxygen, glucose enters the tricarboxylic acid (TCA) cycle and the electron transfer chain, resulting in the formation of 38 molecules of adenosine 5′-triphosphate (ATP). In the absence of adequate oxygen, pyruvate is converted to lactate rather than initiating the TCA cycle, resulting in decreased ATP production. The liver removes half of this lactate, while the kidneys and muscles handle the other half. Lactic acidosis (arterial pH less than or equal to 7.35 with lactate > 5 mmol/l) and sepsis have been connected for more than 200 years, as has tissue hypoxia for more than 100 years [2, 3].

Cohen and Woods have identified two types of hyperlactatemia: type A, which is linked to inadequate tissue oxygenation in illnesses like sepsis, shock, hypovolemia, and extreme hypoxia; and type B, which is seen with sufficient oxygenation and regular hemodynamics and can be associated with certain medical conditions such as liver failure or cancer, medications or toxins, or genetic metabolic disorders [4]. In our case, we attempted to rule out the majority of the lactic acidosis reasons listed above, and the hyperlactatemia was deemed to be type B. Lactic acidosis is recognized as a survival predictor in trauma and sepsis patients, and its normalization during the first 24 hours following the insult favors a favorable result [5]. When lactic acidosis is accompanied by hypoperfusion or sepsis, the mortality rate increases by a factor of nearly three, and the overall result in these patients is directly proportional to the lactate level [6].

According to a research report published in the United States of America, the majority of herbal remedies used are based on extensive clinical and traditional experiences and have not been subjected to safety analyses through the drug-development process [7].

The Roussel Uclaf Causality Assessment Method (RUCAM) is a tool for quantifying causality in cases of suspected drug-induced liver damage (DILI) and herb-induced liver injury (HILI) [8]. The revised RUCAM scoring system provides a wide range of final scores ranging from +14 to -9 points (Table 2).

**Table 2:** Causality assessment scoring by updated RUCAM RUCAM - Roussel Uclaf Causality Assessment Method

Points	Causality
≤0	Excluding causality
1-2	Unlikely
3-5	Possible
6-8	Probable
≥9	Highly probable

The RUCAM-based assessment has a high sensitivity of 86% and specificity of 89% [8]. Our case was evaluated using these criteria, and we discovered that our patient suffered hepatocellular damage. The updated RUCAM score was seven indicating that the causation of this hepatocellular injury was highly likely due to this herbal drug (Appendix). A similar case report published by Teschke et al. elaborated on causality assessment with the updated Council for International Organizations of Medical Sciences scale to show probable causality for an Ayurvedic herbal product causing hepatotoxic reactions [9].

Many people around the world seek traditional alternative medicine to treat their diabetes. Nearly half of the diabetic patients take both oral hypoglycemic medicines and herbal drugs; however, only around 5% of these people consult with an Ayurvedic practitioner before utilizing these herbs [10]. This co-therapy may result in drug-herb interactions, which can take the form of antagonistic or synergistic effects [11,12]. Insulin Management Expert (IME-9) is an anti-diabetes product developed by the Central Council for Research in Ayurvedic Sciences that is composed of five herbs named Aam (*Mangifera indica*), Karela (*Momordica charantia*), Gudmar (*Gymnema Sylvestre*), Jamun (*Syzygium cumini*), and Shudh Shilajit (Asphalt), as described in a review article by Gajarmal, which also mentions a double-blind clinical human trial study of >800 patients, the details of which could not be sought on a thorough literature search with all possible keywords [13]. Furthermore, the official website for IME-9 does not go into detail about its safety profile other than to say that it is safe from side effects and scientifically validated [14]. The scarcity of contemporary data on the safety profile of this over-the-counter drug is concerning. Another study described the efficacy of IME-9 in newly diagnosed type 2 diabetics in a retrospective analysis but did not discuss any negative effects [15].

## Conclusions

Lactate measurement and clearance are critical components in the prognosis and therapy of septic shock patients. Lactic acidosis includes many more differential diagnoses than septic shock and hypoxia, about which we should be very careful before prognosticating such patients, combined with an awareness of lactate metabolism physiology. Although the mechanism is uncertain, according to the RUCAM criteria for HILI and DILI etiology, IME-9 ingestion is associated with hepatotoxicity and hence lactic acidosis, but more research is needed to determine the specific mechanism of this.

## Figures and Tables

**Figure 1:**
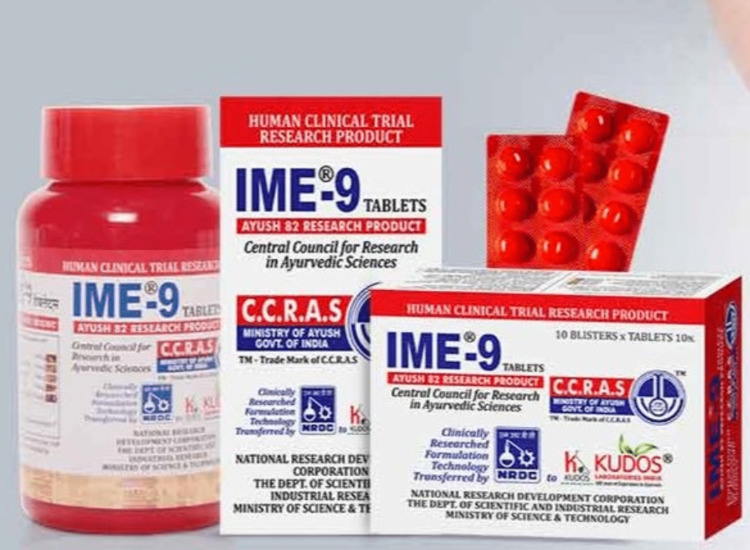
Insulin Management Expert (IME-9) product

**Figure 2:**
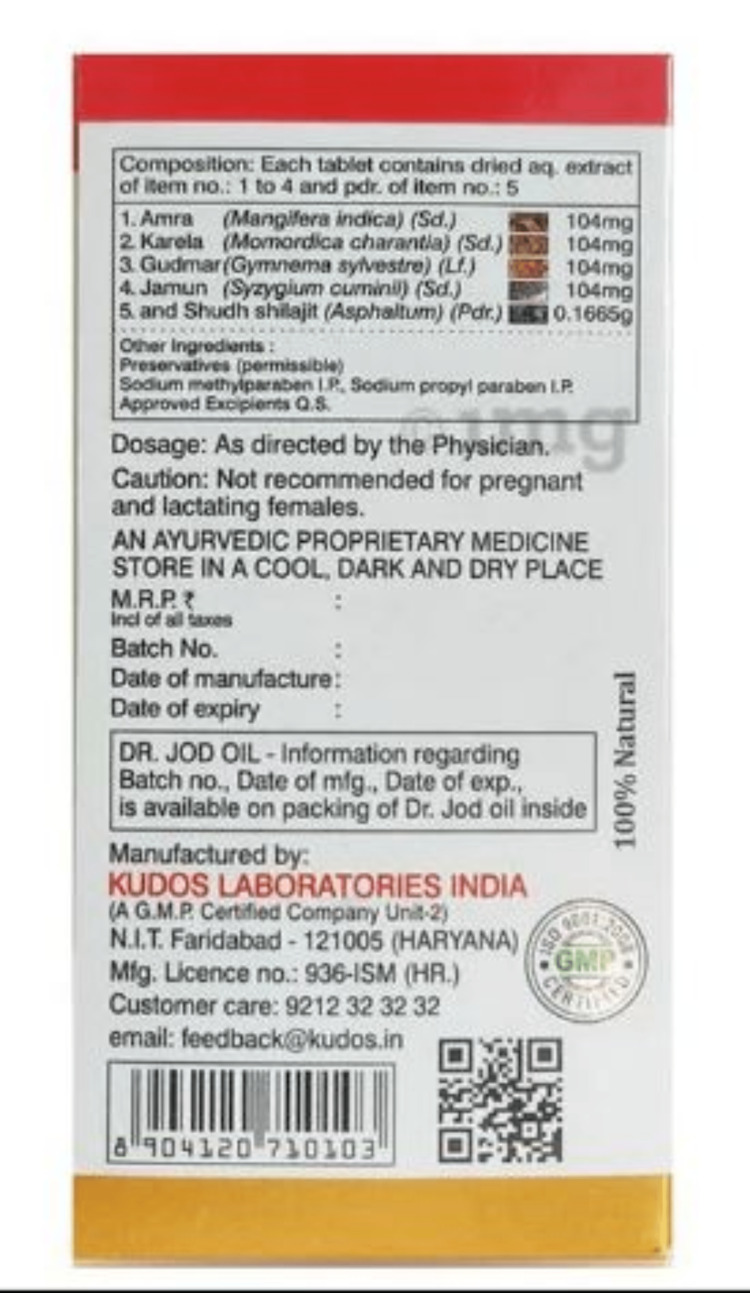
Insulin Management Expert (IME-9) ingredients

**Figure 3:**
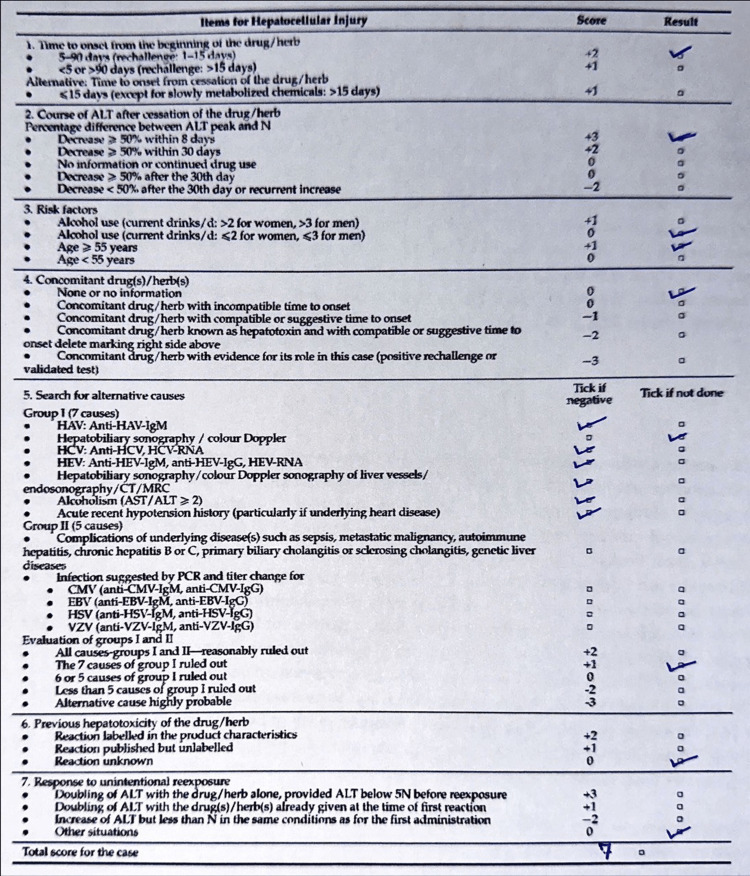
Updated RUCAM scoring sheet RUCAM - Roussel Uclaf Causality Assessment Method
